# Supplementary figures and images for: 5-ALA does not potentiate dihydroartemisinin against Plasmodium falciparum malaria parasites
Source: EMBO Mol Med. 2026 Feb 27;18(4):1130–3. doi: 10.1038/s44321-026-00388-7 (PMC13083901; doi:10.1038/s44321-026-00388-7)

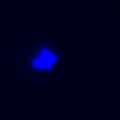

Supplement: Supplementary file 2 — Source data Fig. 1 [file 44321_2026_388_MOESM2_ESM.zip › SourceDataFig1/1D/0-ALA-Alexa568_06-Troph-c-blue.tif]

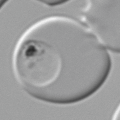

Supplement: Supplementary file 2 — Source data Fig. 1 [file 44321_2026_388_MOESM2_ESM.zip › SourceDataFig1/1D/0-ALA-Alexa568_06-Troph-c-DIC.tif]

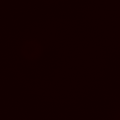

Supplement: Supplementary file 2 — Source data Fig. 1 [file 44321_2026_388_MOESM2_ESM.zip › SourceDataFig1/1D/0-ALA-Alexa568_06-Troph-c-red.tif]

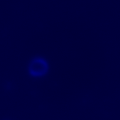

Supplement: Supplementary file 2 — Source data Fig. 1 [file 44321_2026_388_MOESM2_ESM.zip › SourceDataFig1/1D/0-ALA-Alexa568_19-Ring-c-blue.tif]

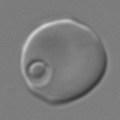

Supplement: Supplementary file 2 — Source data Fig. 1 [file 44321_2026_388_MOESM2_ESM.zip › SourceDataFig1/1D/0-ALA-Alexa568_19-Ring-c-DIC.tif]

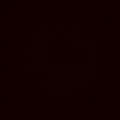

Supplement: Supplementary file 2 — Source data Fig. 1 [file 44321_2026_388_MOESM2_ESM.zip › SourceDataFig1/1D/0-ALA-Alexa568_19-Ring-c-red.tif]

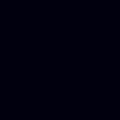

Supplement: Supplementary file 2 — Source data Fig. 1 [file 44321_2026_388_MOESM2_ESM.zip › SourceDataFig1/1D/0-ALA-Alexa568_43-RBC-c-blue.tif]

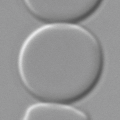

Supplement: Supplementary file 2 — Source data Fig. 1 [file 44321_2026_388_MOESM2_ESM.zip › SourceDataFig1/1D/0-ALA-Alexa568_43-RBC-c-DIC.tif]

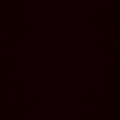

Supplement: Supplementary file 2 — Source data Fig. 1 [file 44321_2026_388_MOESM2_ESM.zip › SourceDataFig1/1D/0-ALA-Alexa568_43-RBC-c-red.tif]

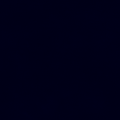

Supplement: Supplementary file 2 — Source data Fig. 1 [file 44321_2026_388_MOESM2_ESM.zip › SourceDataFig1/1D/1000-ALA-Alexa568_54-RBC-c-blue.tif]

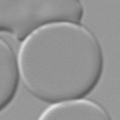

Supplement: Supplementary file 2 — Source data Fig. 1 [file 44321_2026_388_MOESM2_ESM.zip › SourceDataFig1/1D/1000-ALA-Alexa568_54-RBC-c-DIC.tif]

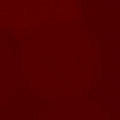

Supplement: Supplementary file 2 — Source data Fig. 1 [file 44321_2026_388_MOESM2_ESM.zip › SourceDataFig1/1D/1000-ALA-Alexa568_54-RBC-c-red.tif]

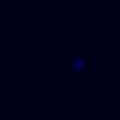

Supplement: Supplementary file 2 — Source data Fig. 1 [file 44321_2026_388_MOESM2_ESM.zip › SourceDataFig1/1D/1000-ALA-Alexa568_58-Ring-c-blue.tif]

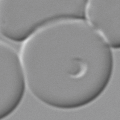

Supplement: Supplementary file 2 — Source data Fig. 1 [file 44321_2026_388_MOESM2_ESM.zip › SourceDataFig1/1D/1000-ALA-Alexa568_58-Ring-c-DIC.tif]

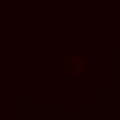

Supplement: Supplementary file 2 — Source data Fig. 1 [file 44321_2026_388_MOESM2_ESM.zip › SourceDataFig1/1D/1000-ALA-Alexa568_58-Ring-c-red.tif]

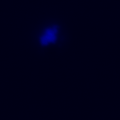

Supplement: Supplementary file 2 — Source data Fig. 1 [file 44321_2026_388_MOESM2_ESM.zip › SourceDataFig1/1D/1000-ALA-Alexa568_62-Troph-c-blue.tif]

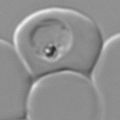

Supplement: Supplementary file 2 — Source data Fig. 1 [file 44321_2026_388_MOESM2_ESM.zip › SourceDataFig1/1D/1000-ALA-Alexa568_62-Troph-c-DIC.tif]

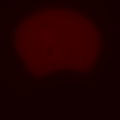

Supplement: Supplementary file 2 — Source data Fig. 1 [file 44321_2026_388_MOESM2_ESM.zip › SourceDataFig1/1D/1000-ALA-Alexa568_62-Troph-c-red.tif]

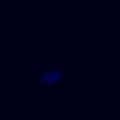

Supplement: Supplementary file 2 — Source data Fig. 1 [file 44321_2026_388_MOESM2_ESM.zip › SourceDataFig1/1D/200-ALA-Alexa568_37-Ring-c-blue.tif]

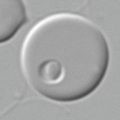

Supplement: Supplementary file 2 — Source data Fig. 1 [file 44321_2026_388_MOESM2_ESM.zip › SourceDataFig1/1D/200-ALA-Alexa568_37-Ring-c-DIC.tif]

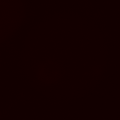

Supplement: Supplementary file 2 — Source data Fig. 1 [file 44321_2026_388_MOESM2_ESM.zip › SourceDataFig1/1D/200-ALA-Alexa568_37-Ring-c-red.tif]

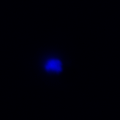

Supplement: Supplementary file 2 — Source data Fig. 1 [file 44321_2026_388_MOESM2_ESM.zip › SourceDataFig1/1D/200-ALA-Alexa568_48-Troph-b-blue.tif]

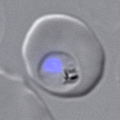

Supplement: Supplementary file 2 — Source data Fig. 1 [file 44321_2026_388_MOESM2_ESM.zip › SourceDataFig1/1D/200-ALA-Alexa568_48-Troph-b-DIC.tif]

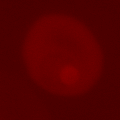

Supplement: Supplementary file 2 — Source data Fig. 1 [file 44321_2026_388_MOESM2_ESM.zip › SourceDataFig1/1D/200-ALA-Alexa568_48-Troph-b-red.tif]

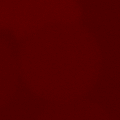

Supplement: Supplementary file 2 — Source data Fig. 1 [file 44321_2026_388_MOESM2_ESM.zip › SourceDataFig1/1D/200-ALA-Alexa568_49-RBC-b-red.tif]

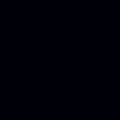

Supplement: Supplementary file 2 — Source data Fig. 1 [file 44321_2026_388_MOESM2_ESM.zip › SourceDataFig1/1D/200-ALA-Alexa568_49-RBC-c-blue.tif]

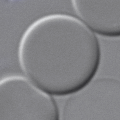

Supplement: Supplementary file 2 — Source data Fig. 1 [file 44321_2026_388_MOESM2_ESM.zip › SourceDataFig1/1D/200-ALA-Alexa568_49-RBC-c-DIC.tif]
